# Care Home Research: Future Challenges and Opportunities

**DOI:** 10.3390/geriatrics4010002

**Published:** 2018-12-21

**Authors:** Alexandra M. Johnstone, Alison I. C. Donaldson

**Affiliations:** On behalf of the Ageing Gut Brain (Age-GB) Study Team, University of Aberdeen, Foresterhill Health Campus, Aberdeen AB25 2ZD, UK; alison.donaldson@abdn.ac.uk

The historical view of care homes as isolated communities is fading, with a new strong focus on offering person-centred care for residents that keeps them integrated in their community [1,2]. This is a welcome approach with the demographical change in our population resulting in a greater number of older adults requiring residential care. Integration of care home residents with their local community may be approached by ‘going out’ such as on excursions to entertainment venues, or an alternative approach is to ‘bring the outside in’ such as inviting local children and groups to participate in mutual activities with residents [3]. A more radical approach is the concept of ‘intergenerational care’ that combines pre-school care with that for residents in care homes, such as has been propelled into the limelight by the UK Channel 4 documentary ‘Old People’s Home for 4 Year Olds’ (https://www.channel4.com/programmes/old-peoples-home-for-4-year-olds).

Adopting an approach of inclusion in society for care home residents may seem right and the obvious mode of care to follow, but this vulnerable group are often under-represented in research studies. It is interesting to consider whether this is because funders and researchers in the past have incorrectly assumed that it is too difficult to include care home residents, or whether there have been genuine ethical concerns for involving this group in research? An integrative approach recognises that people living in care homes may have a wish to be involved in research that offers an opportunity to ‘make a difference’ for those following in their footsteps, and moreover that they may enjoy the interaction with researchers, and the sense of achievement from contributing [4]. This can be achieved to facilitate innovative research in tandem with the rights, safety and dignity of participants considered.

If we strive to improve quality of life for care home residents, then we need high quality evidence to help drive changes in practice and improve care standards. For this we must consider how researchers can support care homes to become involved with studies, including trials, and how they can be designed in a way to minimise disruption to residents and staff [5].

A current study giving care home residents the opportunity to participate in research is the ‘Ageing Gut Brain Study’ (AGE-GB) at the University of Aberdeen, which seeks to understand the role of the gut microbiota in Alzheimer’s dementia. The role of gut microbiome interacts with the brain in a bi-directional manner [6] with focused research to determine the mechanistic pathways to develop strategies for prevention or treatment [6]. We clearly need to understand the nature of that impact, the underlying mechanisms, and how changes in diet can reprogram our gut microbiota–brain axis to resolve or reduce clinical symptoms associated with dementia. There is increasing evidence that identifies the gut microbiota as a key conduit between nutrition and brain function where the composition of the microbiota correlates with diet and health in the elderly [7]. The hypothesis of AGE-GB is that the gut–brain axis plays a key role in behavioural changes in dementia and that it is responsive to changes in the gut microbiota profile.

Perhaps one of the greatest challenges to care home research is the careful consideration needed for those with cognitive impairment and how to ensure inclusion in research for those who cannot give informed consent, but whose wishes would be to participate [8]. The survey conducted by the AGE-GB study found that the median percentage (IQR) of care home residents with any type of dementia was 70.2% (51.3–84.7), and the median percentage (IQR) of those with dementia with challenging behaviours was 34.8% (17.4–50.0). Clearly, if we are to perform meaningful and inclusive research in the care home environment that drives forward improvements in care standards and quality of life for this vulnerable group, then there needs to be support from residents and their families, care home staff, and ethics boards. Participation for those with dementia and no longer able to give informed consent can be made possible by recognising their previously expressed wishes in a process of consent by proxy.

Successful research in care homes requires a partnership that gives residents a voice, ensures the care home benefits from participating and that the researcher has the required support. A hospital in-patient no longer able to manage independently at home recently quoted to me in a conversation about discharge plans that ‘East, West, home best’ meaning that she had reservations about whether she could be happy as a resident in a care home; it is our challenge as researchers to overcome any hurdles and improve the quality of life and perceptions of residential care to ensure that people can confidently ‘age well’ in the care home environment.
